# Regulation of wound ethylene biosynthesis by NAC transcription factors in kiwifruit

**DOI:** 10.1186/s12870-021-03154-8

**Published:** 2021-09-08

**Authors:** Niels J. Nieuwenhuizen, Xiuyin Chen, Mickaël Pellan, Lei Zhang, Lindy Guo, William A. Laing, Robert J. Schaffer, Ross G. Atkinson, Andrew C. Allan

**Affiliations:** 1 The New Zealand Institute for Plant and Food Research Limited (PFR), Private Bag 92169, Auckland, 1142 New Zealand; 2 School of Biological Sciences, University of Auckland, Private Bag 92019, Auckland, 1142 New Zealand; 3 Institute of Fruit and Tea, Hubei Academy of Agricultural Sciences, Wuhan, 430064 China; 4 PFR, Private Bag 11600, Palmerston North, 4442 New Zealand; 5 PFR, 55 Old Mill Road, RD 3, Motueka, 7198 New Zealand

**Keywords:** Kiwifruit, Wounding, Ethylene, Biosynthesis, Regulation, NAC, Transcription factors

## Abstract

**Background:**

The phytohormone ethylene controls many processes in plant development and acts as a key signaling molecule in response to biotic and abiotic stresses: it is rapidly induced by flooding, wounding, drought, and pathogen attack as well as during abscission and fruit ripening. In kiwifruit (*Actinidia* spp.), fruit ripening is characterized by two distinct phases: an early phase of system-1 ethylene biosynthesis characterized by absence of autocatalytic ethylene, followed by a late burst of autocatalytic (system-2) ethylene accompanied by aroma production and further ripening. Progress has been made in understanding the transcriptional regulation of kiwifruit fruit ripening but the regulation of system-1 ethylene biosynthesis remains largely unknown. The aim of this work is to better understand the transcriptional regulation of both systems of ethylene biosynthesis in contrasting kiwifruit organs: fruit and leaves.

**Results:**

A detailed molecular study in kiwifruit (*A. chinensis*) revealed that ethylene biosynthesis was regulated differently between leaf and fruit after mechanical wounding. In fruit, wound ethylene biosynthesis was accompanied by transcriptional increases in 1-aminocyclopropane-1-carboxylic acid (ACC) synthase (ACS), ACC oxidase (ACO) and members of the NAC class of transcription factors (TFs). However, in kiwifruit leaves, wound-specific transcriptional increases were largely absent, despite a more rapid induction of ethylene production compared to fruit, suggesting that post-transcriptional control mechanisms in kiwifruit leaves are more important. One ACS member, *AcACS1*, appears to fulfil a dominant double role; controlling both fruit wound (system-1) and autocatalytic ripening (system-2) ethylene biosynthesis. In kiwifruit, transcriptional regulation of both system-1 and -2 ethylene in fruit appears to be controlled by temporal up-regulation of four NAC (NAM, ATAF1/2, CUC2) TFs (*AcNAC1–4*) that induce *AcACS1* expression by directly binding to the *AcACS1* promoter as shown using gel-shift (EMSA) and by activation of the *AcACS1* promoter *in planta* as shown by gene activation assays combined with promoter deletion analysis.

**Conclusions:**

Our results indicate that in kiwifruit the NAC TFs AcNAC2–4 regulate both system-1 and -2 ethylene biosynthesis in fruit during wounding and ripening through control of *AcACS1* expression levels but not in leaves where post-transcriptional/translational regulatory mechanisms may prevail.

**Supplementary Information:**

The online version contains supplementary material available at 10.1186/s12870-021-03154-8.

## Background

The phytohormone ethylene controls many processes in plant development and acts as a key signaling molecule in response to biotic and abiotic stresses: it is rapidly induced by stress signals such as flooding, wounding, drought, and pathogen attack [1–3], as well as during other important physiological processes such as abscission, reproductive biology and fruit ripening [4–8]. Ethylene regulates its own biosynthesis through positive and negative feedback loops [9–12] leading to the proposal of two systems of ethylene regulation [13]. System-1 ethylene is auto-inhibitory and associated with low amounts of ethylene. When ethylene is induced by wounding or pathogens, it is rapidly down-regulated. System-2 is autocatalytic and occurs during fruit ripening and during petal senescence in some species [14, 15], and is often accompanied by an increase in respiration (a “climacteric rise”).

Ethylene is synthesized from the amino acid methionine, which is converted to *S*-adenosylmethionine (SAM) by SAM-synthase. SAM is converted to 1-aminocyclopropane carboxylic acid (ACC) and 5′-methylthioadenosine (MTA) by the enzyme ACC synthase (ACS) [16]. The final step is the conversion of ACC to ethylene by ACC oxidase (ACO) [17]. Adding the substrate ACC to plants creates ethylene [18–20], suggesting that the key regulatory step in ethylene biosynthesis is controlled by ACS [12, 17, 21]. ACS is regulated at the transcriptional and post-transcriptional level in plants. In contrast, ACO is generally constitutively expressed in system-1, but strongly induced in system-2 and therefore may only be limiting late in ripening [22–26].

**Fig. 1:**
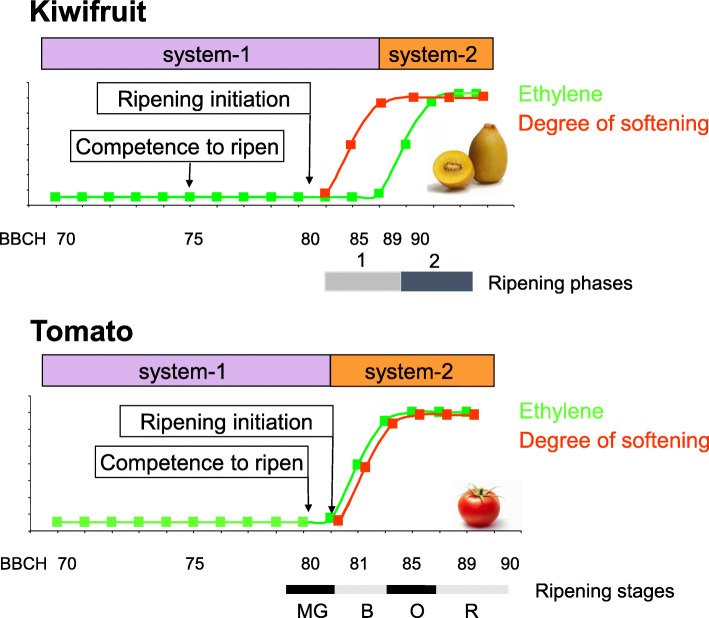
Different models of ripening behavior in kiwifruit and tomato. In kiwifruit, the competence to ripen occurs well before ripening initiation. Ripening initiation and the initial softening period (ripening phase 1) are accompanied by non-autocatalytic ethylene production (system-1) and are separated from the late ripening period (ripening phase 2) that is accompanied by autocatalytic ethylene production (system-2). Kiwifruit ripening stages are defined in Richardson et al. (2011) [27] and tomato ripening stages in Feller et al. (1995) [28]. The BBCH plant development scale is described in Hess et al. (1997) [29]. In tomato, the competence to ripen (responsiveness to exogenous ethylene) coincides with the mature green (MG) stage and is closely followed by ripening initiation and autocatalytic ethylene production and softening. MG: mature green, B: breaker, O: orange, R: red

One of the best studied areas of ethylene regulation is during tomato (*Solanum lycopersicum*) fruit development and ripening (Fig. 1). Tomato fruit progresses through well-defined growth stages, ripening through a progression from mature green, breaker, orange and red ripe [28]. The mature green to breaker stage is associated with a switch from system-1 to system-2 ethylene during which the fruit rapidly soften, undergo a color change and an increase in aroma volatiles. Kiwifruit (*Actinidia* spp.) fruit development has also been well characterized, with defined growth progression through a Biologische Bundesanstalt, Bundessortenamt und Chemische Industrie (BBCH, [29]) scale of fruit growth and development [27, 30]. Fruit maturation occurs at BBCH 80. After an initial period of system-1 ripening (phase 1) that is associated with starch break down, softening and color change then ensues, followed by a period of system-2 (phase 2) ripening (at BBCH 90) associated with production of aroma volatiles and further softening. At BBCH 80, there is a progressive ability to ripen with exogenous ethylene or propylene. However, endogenous ethylene production is repressed in phase 1 ripening. The *AcACS1* gene is associated with system-1 and -2 ripening, and its expression could only be induced transiently with exogenous propylene treatment during phase 1 ripening. Once phase 2 ripening started, *AcACS1* was no longer repressed [30].

Wounding in both leaves and fruit in plants is associated with a number of signals that occur in parallel and in sequence over time and in space. In Solanaceae upon wounding, the small peptide systemin [31] acts as an early local and systemic signal while reactive oxygen species (ROS) [32] and oligosaccharides such as oligogalacturonides have also been identified as early wound signals in several plant species in combination with certain receptor signaling pathways that may detect cell wall integrity [33, 34]. Other rapid signals include electrical signals [35], ion fluxes [36] and MAP kinase signalling pathways [37, 38]. Apart from the plant hormones jasmonic acid (JA) and abscisic acid (ABA), ethylene has also been implicated in the wounding process acting as a local and systemic signal [16, 39, 40] and is involved in the cross-talk between various wound signaling pathways [41]. In mature green tomato fruit, wounding resulted in rapid induction of ethylene and *SlACS1A* and *6* within 30 min. In wounded leaves, *SlACS1A* and *6* induction could be detected within 10 min [42], with expression of both genes returning to baseline levels after 4 h. In kiwifruit, very little is known about wound ethylene production. Studies on the effect of brushing kiwifruit to remove fruit trichomes, and the effect of mechanical impact injury on ripening behavior of fruit, showed that both treatments accelerated ripening during subsequent storage of fruit and were accompanied by increases in ethylene production, soluble solid concentration and decreased firmness [43, 44]. In both cases, wound ethylene produced from the fruit skin was the likely cause of accelerated ripening. *AcACO1* RNAi silenced kiwifruit lines produced no wound ethylene in leaves as well as no detectable levels of climacteric ethylene in fruit [45], suggesting *AcACO1* is a major gene involved in both fruit and leaf ethylene production.

Multiple transcription factor (TF) families have been implicated in the control of ripening and ethylene transcriptional regulation in fruit and leaves. In tomato, a MADS box centric positive feedback loop for climacteric ethylene (system-2) has been presented consisting of RIN, TAGL1 and ACS2, while in other climacteric fruit species either NAC or mixed MADS/NAC positive feedback loops were identified; all these loops include the ethylene stabilized EIN3 (ethylene-insensitive3) TF [46] but MYB transcription factors have also been implicated in regulating ethylene biosynthesis [47, 48]. Recent work on CRISPR knockout lines has redefined the role of wildtype RIN, NAC-NOR, and SBP-CNR during ripening in tomato [49–51] as these mutant lines showed more subtle phenotypes. In kiwifruit *(A. chinensis*/*A. arguta*), NAC TF expression of several family members is highly induced during late fruit ripening in concert with autocatalytic ethylene production and induces ripening associated terpene synthases [52] and is also associated with ethylene production under control of micro RNA 164 (miR164) [53] and/or low temperature induced ripening [54]. A SEP4/RIN-like MADS box gene has also been implicated in regulating ethylene biosynthesis in ripe fruit [30]. In *A. deliciosa* kiwifruit methyl jasmonate treatment of fruit could stimulate ethylene production beyond ethylene treatment alone and two NAC transcription factors were implicated in the increased ACS induction in ripening fruit [55].

While the involvement of NAC TFs in regulating fruit ripening/system-2 ethylene is well established in certain species, less is known about system-1 wounding related ethylene production and control in fruit and leaves. In this study, we examined the relationship between system-1 and -2 ethylene production in wounded fruit and leaves of kiwifruit (*A. chinensis*) and the involvement of four NAC TFs in gene regulation through promoter interaction of ethylene biosynthesis genes. Our aim was to investigate how ethylene production is regulated and identify potential conservation of control mechanisms across different organs and ethylene systems.

## Results

### Ethylene production in kiwifruit after mechanical wounding of immature fruit

**Fig. 2:**
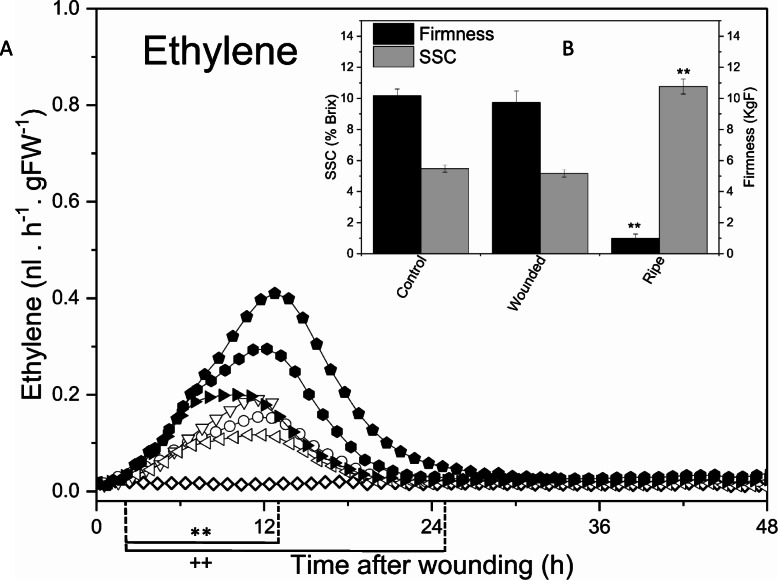
Ethylene production in wounded immature *A. chinensis* ‘Hort16A’ kiwifruit. (**A**) Fruit were wounded with two incisions (open symbols) or four incisions (closed symbols) and transferred to a 1.5 L sealed jar at 2 L h^− 1^ air flow. Ethylene production was measured continuously over the time course shown. Control unwounded fruit (open diamond) produced no ethylene over the course of the experiment. Three biological repeats (consisting of three fruit each) were harvested per time point from fruit with two incisions. (**B**) Soluble solids concentration (SSC, % Brix) and firmness (kgF) changes in immature fruit after wounding. Control: unwounded fruit; Wounded: fruit cut with 2 incisions were measured at 120 h after wounding; Ripe: eating ripe fruit. Data are the mean ± SE, n = nine biological replicates. **/++: statistically different compared to control in two/four cut respectively (*p* < 0.01, ANOVA (**A**) /Student’s t-test (**B**)

Immature *A. chinensis* var. *chinensis* ‘Hort16A’ kiwifruit (BBCH 78 [29], ~ 80% of final weight with seeds about to start turning black) harvested at 110 days after full bloom (DAFB) [27], and demonstrating a system-1 ethylene response [30] upon exogenous ethylene treatment, were wounded with two or four incisions after which ethylene production was monitored over 48 h and compared to eating ripe fruit. All wounding resulted in a transient burst of ethylene that peaked at around 12 h after wounding (Fig. 2A) with fruit with four incisions producing approximately twice as much ethylene compared to fruit with two incisions as is expected due to the increased wound surface. The ethylene levels then rapidly declined to near baseline levels at 24 h after wounding, characteristic of system-1 ethylene production. In the cut fruit, the soluble sugars and firmness did not change significantly over the 120 h assessed (Fig. 2B) and no detectable ethylene was produced post 24 h. In contrast, eating ripe fruit were soft with high levels of SSC and produced much higher levels of (autocatalytic) ethylene compared to wounded fruit (> 100-fold more).

### Comparison of the tomato and kiwifruit ACS genes

**Fig. 3:**
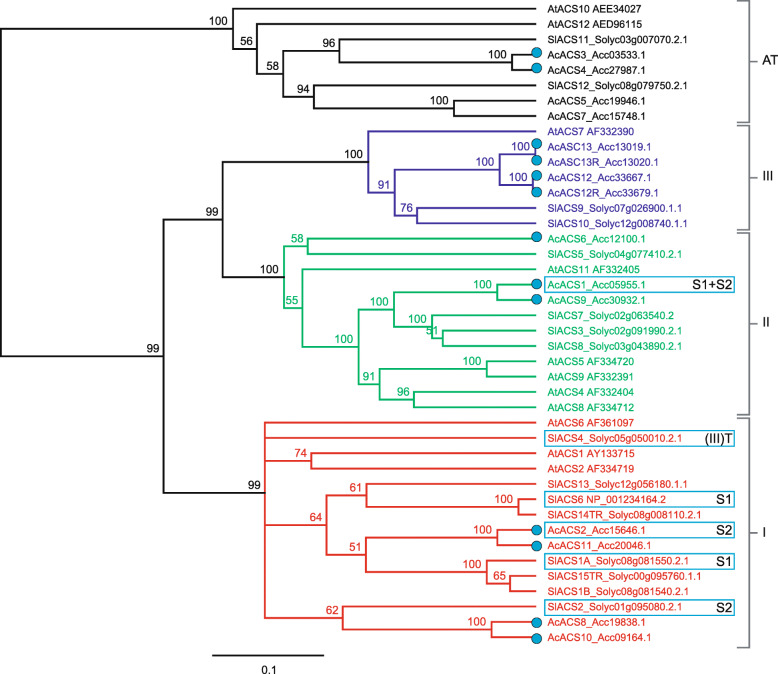
Consensus phylogram of aminocyclopropane-1-carboxylic acid synthase (ACS) proteins from Arabidopsis, tomato and kiwifruit. Predicted ACS proteins from Arabidopsis (At), tomato (Sl), and kiwifruit (Ac/blue dot) were aligned using the Geneious MUSCLE alignment tool and a consensus UPGMA bootstrap phylogram was generated using 1000 replicates (Jukes-Cantor distance matrix). Only branches with over 50% support threshold are displayed. AT = aminotransferase. Type I, II and III ACS proteins were assigned based on presence of conserved C-terminal sequences (see Supplemental Fig. S1 for alignment). Type I (red) = RLSF/SLSF only; type II (green) = WVF, RLSF and RDE rich domains (TOE/target of ETO1 domain); type III (blue) = absence of type I/II domains (based on Yoshida et al. 2006) [57]. (III) = Both SlACS4 and 13 cluster with type I, but show absence of typical type I residues in the C-terminus (see Supplemental Fig. S1 for alignment). S1, S2 = involved in system-1, − 2 ethylene production. T = involved in transition between system-1 and -2

Fifteen kiwifruit and fourteen tomato ACS genes previously described [7, 56] were aligned to identify those involved in ethylene biosynthesis (Fig. 3). Four kiwifruit ACS-like genes (*AcACS3–5, 7*) and two tomato genes (*SlACS11, 12*) clustered with aminotransferases (AT) [58] and are therefore not likely to be involved in ethylene biosynthesis. Based on the C-terminal sequences, four kiwifruit genes clustered with type I ACS proteins (*AcACS2, 8, 10, 11*), three with type II *(AcACS1, 6, 9*) and four with type III ACS proteins (two pairs of paralogs: *AcACS12/12R* and *13/13R*) (for C-terminal alignments, see Supplemental Fig. S1). In tomato, eight ACS-proteins clustered with type I (Fig. 3, *SlACS1A*, *1B*, *2*, *4*, *6*, *13–15*). Four (*SlACS3*, *5*, *7*, *8*) cluster with type II and two (*SlACS9* and *10)* with type III and show characteristic C-termini (Fig. 3, Supplemental Fig. S1). *SlACS15* (type I) is likely a truncated protein (333 AA in length), while *SlACS4* and *14* (101 AA, truncated) are missing characteristic type I C-terminal residues (Supplemental Fig. S1).

### Expression of ethylene biosynthetic genes after mechanical wounding of immature fruit

**Fig. 4:**
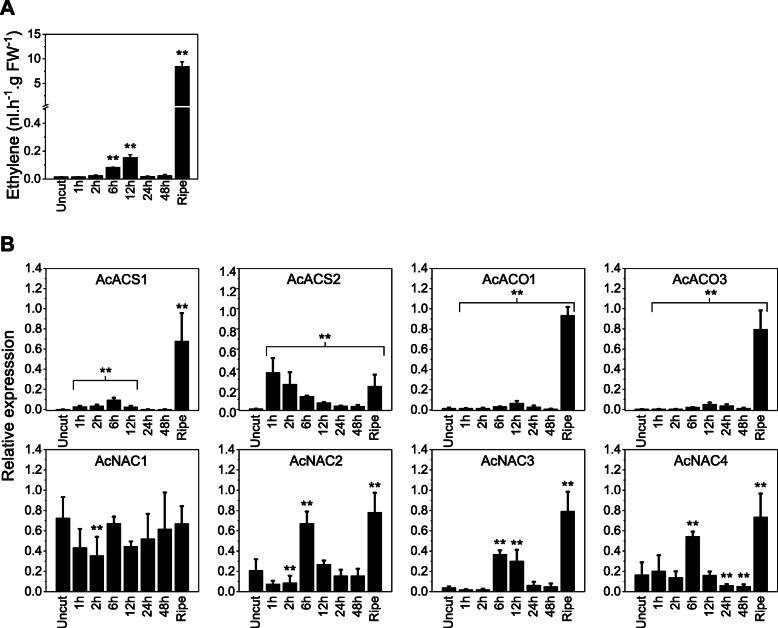
(**A**) Ethylene production after mechanical wounding of immature *A. chinensis* ‘Hort16A’ fruit. Time course as per Fig. 2A with ripe fruit for comparison. (**B**) qRT-PCR expression analysis of ethylene biosynthetic (ACS, ACO) genes and NAC TFs in wounded fruit. Data are mean ratio calibrated ± SD, n = three biological replicates and expressed as a ratio compared to the *PP2A* reference gene. **: *p* < 0.01 significantly different compared to unwounded fruit, based on mixed model statistics (R packages nlme and emmeans, as described in Materials and Methods

Quantitative reverse transcription polymerase chain reaction (qRT-PCR) analysis was performed to determine the expression of ACS genes in the immature kiwifruit wounding experiment (excluding the four AT members) over the 48 h time course from RNA extracted at 0, 1, 2, 6, 12, 24, 48 h after wounding. *AcACS1* showed the highest induction of expression during wounding (system-1) and expression preceded the release of wound ethylene and was much lower than in ripe fruit (Fig. 4). *AcACS2* showed rapid induction in the wounding phase but overall peak transcript levels were estimated to be > 10 fold lower than *AcACS1* (Supplemental Table S1 - ratio) during the wound ethylene production phase. The other eight ACS genes showed low overall levels of transcription during the experiment (Supplemental Table S1).

Expression of nine ACO genes previously identified in the kiwifruit genome [56] was also monitored after mechanical wounding of ripe fruit. Expression of *AcACO1* and *AcACO3* closely mirrored ethylene levels throughout the time course, peaking at 12 h after wounding (system-1) and overall showed highest expression in ripe fruit (system-2). The seven other ACO genes showed either lower levels of expression or no expression during the time course compared to *AcACO1* and *AcACO3* (Supplemental Table S1).

### Kiwifruit NAC TFs involved in system-1 and -2 ethylene biosynthesis

**Fig. 5:**
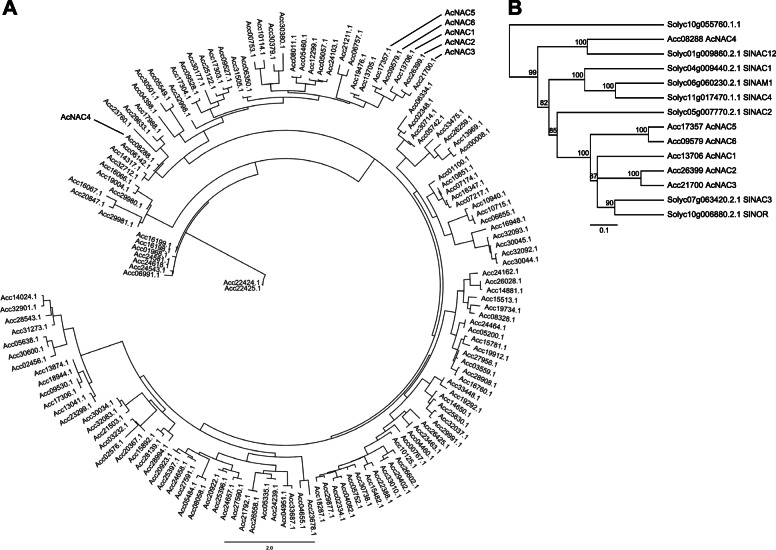
Phylogenetic analysis of putative NAC transcription factors from kiwifruit. (**A**) The 147 putative NAC TFs identified in the *A. chinensis* ‘Red5’ genome (see Supplemental Table S2) were initially aligned using Geneious MUSCLE alignment tool, then manually curated. The DNA binding site was extracted and realigned using Muscle. Phyml [59] was used to construct the tree shown using the JTT (Jones, Taylor & Thornton) substitution method with default calculation parameters and was rooted with Acc22424.1 and Acc22425.1 as outgroup. (**B**) A UPGMA consensus tree of kiwifruit (Ac) and tomato (Solyc) NAC TFs (complete ORFs). The tree was generated using 1000 replicates (Jukes-Cantor distance matrix). Only branches with over 50% support threshold are displayed. Solyc10g55760 is used as outgroup

Analysis of the kiwifruit genome identified 147 putative NAC family members in the kiwifruit genome (Fig. 5; Supplemental Table S2). The kiwifruit ripening-related *AcNAC1–3* genes previously described [52] all clustered closely with *SlNOR* (*LeNOR)* and *SlNAC3* (Fig. 5) and showed conservation over the entire length of the protein including a C-terminal “WYS” tail that is also present in Arabidopsis NARS1 and NARS2 (NAM) proteins [60] involved in embryogenesis. Two other kiwifruit genes clustered with *SlNOR* (Fig. 5) (Acc17357 and Acc09579, hereafter named *AcNAC5* and *6* respectively) but did not have the conserved C-terminus (rather WNL/WNS respectively). *AcNAC4* clustered with *SlNAC12* (Solyc01g009860.1/SGN-U563196) in a separate group of proteins of much shorter length (< 300 amino acids) and without the conserved C-terminus, while *SlNAC2* [61] is positioned in between both groups (Fig. 5).

*AcNAC2–4* expression showed a peak at 6 h after wounding, followed by a rapid decline. The expression of *AcNAC2–4* closely mirrored the induction of *AcACS1* after wound treatment (Fig. 4B). *AcNAC1* was essentially constitutively expressed at a high level in unwounded/wounded fruit and only showed a drop in expression at the 2 h time point. *AcNAC5* and *AcNAC6* also showed some induction during wounding, peaking at 6 h after wounding and again in ripe fruit (Supplemental Table S1 - ratio), but at lower transcript levels. Peak fruit expression of *AcNAC2–6* and ethylene levels were all highest in ripe fruit (Fig. 4A/B).

### Ethylene production and gene expression analysis after wounding in kiwifruit leaves

**Fig. 6:**
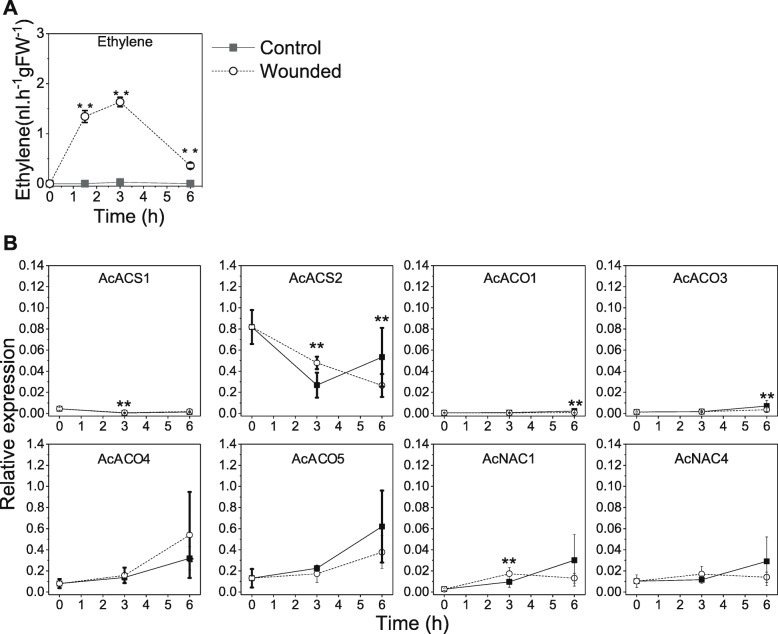
(**A**) Ethylene production in wounded kiwifruit leaves. Expanding *A. chinensis* ‘Hort16A’ leaves were wounded with a 96-well pin blotter and ethylene production was measured continuously over the time course shown (0, 1.5, 3, 6 h after wounding). Data are mean ± SD, *n* = 3 biological replicates. (**B**) qRT-PCR of ethylene biosynthetic (ACO and ACS) genes and NAC TFs in wounded leaves and control leaves (0, 3, 6 h after wounding). Data are mean ratio calibrated ± SD, n = three biological replicates and expressed as a ratio compared to the *PP2A* reference gene. **: *p* < 0.01 significantly different compared to unwounded leaves, based on Student’s t-test (**A**) or mixed model statistics (**B**) (R packages nlme and emmeans, as described in the Materials and Methods)

*AcACO1* RNAi silenced kiwifruit lines produced no wound (system-1) ethylene in leaves and no detectable levels of system-2 ethylene in ripe fruit [45]. These results suggest a link between fruit and leaf ethylene production, where both depend on *AcACO1* expression. To investigate the regulation of system-1 ethylene production in kiwifruit leaves, expanding leaves were wounded by mechanical penetration using a 96-well “pin tool”. In contrast to fruit, ethylene release peaked much more rapidly, at around 3 h post wounding and returned to near baseline after 6 h (Fig. 6A). qRT-PCR analysis indicated that there was little significant up-regulation of ACS, ACO or NAC gene expression after leaf wounding (Fig. 6B and Supplemental Table S1), suggesting that the induction of wound ethylene in kiwifruit leaves does not involve increased transcription of ethylene biosynthetic genes or upstream NAC TFs, but is more likely to be controlled by other steps upstream, or at the post-transcriptional or translational level. Compared to fruit, *AcACO1* and *3* showed lower expression levels, while *AcACO4* and *5* showed higher levels of expression in leaf (Supplemental Table S1, ratio).

### NAC transcription factors in *A. chinensis* activate the ACS promoter

**Fig. 7:**
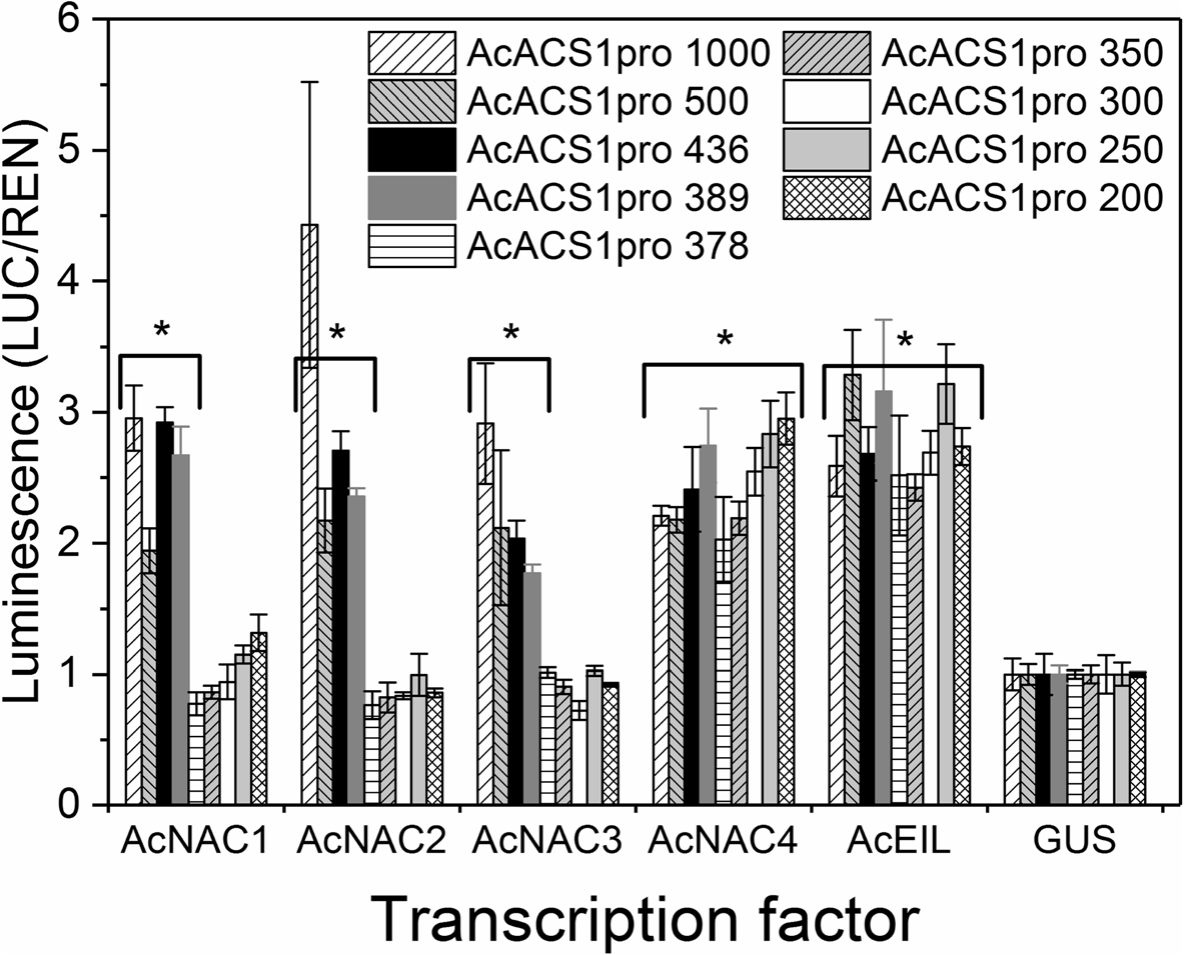
Promoter activation by NAC (NAM, ATAF1/2, CUC2) and EIL (Ethylene-insensitive3-like) TFs using deletions of the *A. chinensis ‘*Hort16A’*AcACS1* promoter. Different sized *AcACS1* regulatory regions (< 1000 bp) upstream of the ATG (AcACS1pro + length in bp) were cloned upstream of the LUC reporter gene of pGreenII-0800LUC in frame with the start ATG and tested for transient activation in *N. benthamiana*. LUC/REN luminescence ratio values of transcription factors *AcNAC1*-*AcNAC4* and a pool of *AcEIL1–4* (equal mixture of *AcEIL1–4*) were compared to a GUS control construct which was set to 1. Statistical differences were determined by Tukey’s honest significant difference test (HSD) after analysis of variance (ANOVA) analysis compared to GUS. Data are mean ± SE, n = three biological replicates (plants), * different at *p* < 0.05

A strong correlation between *AcNAC2–4* and *AcACS1* gene expression in kiwifruit suggested that NAC TFs might directly activate the *AcACS1* promoter in both wounded fruit (system-1) and during ripening (system-2) kiwifruit. A 1 kb fragment of the *AcACS1* upstream region was amplified from *A. chinensis* ‘Hort16A’ genomic DNA and a promoter deletion series was analyzed using the firefly luciferase reporter gene system [62]. Significant activation of the *AcACS1* promoter was observed by AcNAC1, 2, 3 and 4 TFs when tested individually and by a pool of four AcEIL1–4 (AcEIN3-like) TFs using promoter fragments of 1000, 500, 436 and 389 bp (Fig. 7) upstream of the ATG. No activation was observed for AcNAC1–3 combined with shorter promoter fragments of 378, 350, 300, 250 or 200 bp when compared to the GUS control construct. In contrast, activation of the *AcACS1* promoter by AcNAC4 and the AcEIL1–4 pool was observed with all these smaller fragments. These data indicate that a binding site for AcNAC1–3 is present around 384 bp upstream and additional proximal binding sites for AcNAC4 and AcEIL may exist.

### NAC TFs directly bind to the kiwifruit AcACS1 promoter

**Fig. 8:**
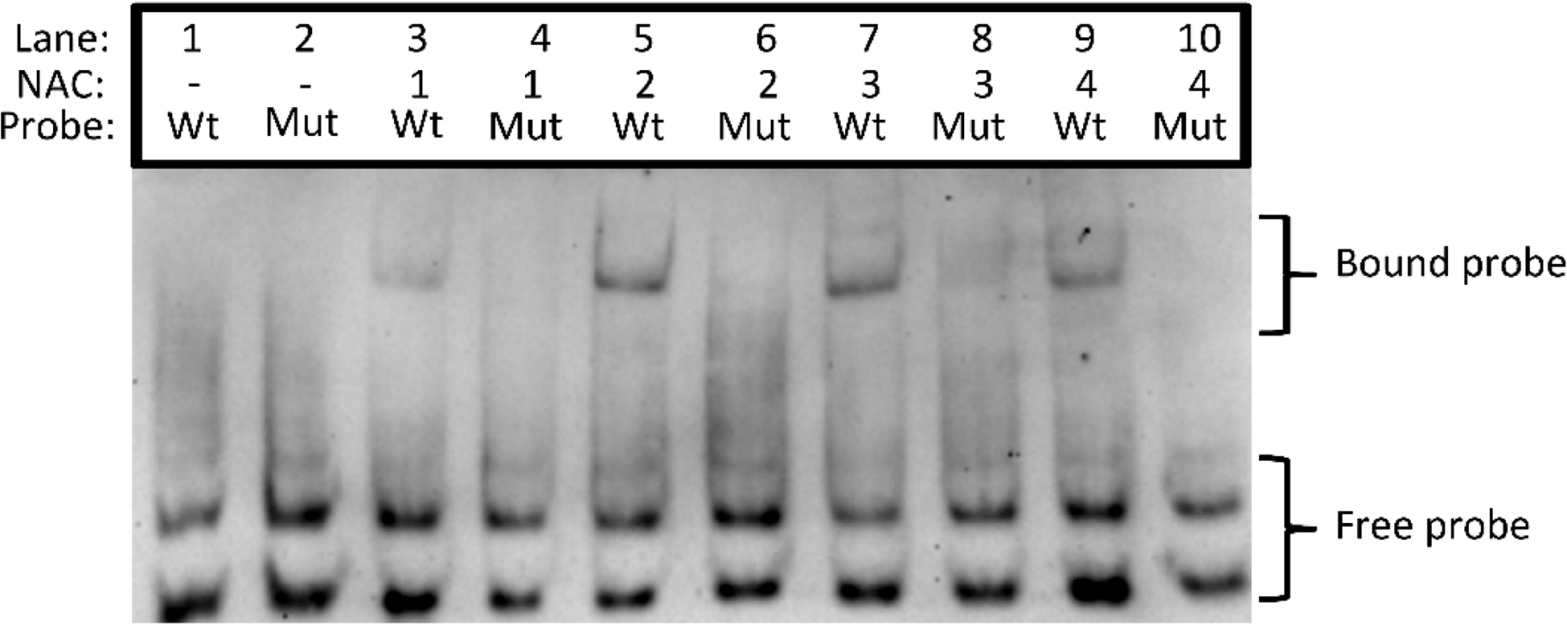
Electrophoretic mobility shift assays (EMSA) of *AcACS1* promoter fragments (27 bp) with recombinant NAC1–4 proteins. The DNA binding domains of NAC1–4 (described in Nieuwenhuizen et al. 2015 [52]) were over-expressed in *Escherichia coli* as Maltose Binding Protein (MBP)-tagged fusion proteins and purified by amylose resin affinity purification and EMSA was run according to Nieuwenhuizen et al. (2015) [52]. Wt = wildtype double-stranded DNA probe with putative NAC palindromic binding site (underlined): CATTATACGTATAGTCAACCACATAAC. Mut = mutated double-stranded DNA probe with randomly mutated NAC binding site (italic/underlined): CAT*CGATCCATCT*GTCAACCACATAAC. NAC = MBP-NAC1, −NAC2, −NAC3 or -NAC4

The kiwifruit *AcACS1* activation study suggests that a NAC DNA binding site may be present between 389 and 378 bp upstream of the ATG. To further investigate this potential binding site, an EMSA (electrophoretic mobility shift assay)/gel shift was performed using wildtype (Wt) and mutated (Mut) promoter fragments (where the putative palindromic NAC binding site TATACGTATA was randomly mutated) surrounding this site (Fig. 8, Supplemental Fig. S2). Double-stranded wildtype and mutated biotin labelled probe migrated rapidly through the gel matrix in the absence of NAC protein (Fig. 8 — free probe, lanes 1, 2). The Wt probe was significantly retarded when incubated with purified NAC1, NAC2, NAC3 and NAC4 proteins (bound probe, lanes 3, 5, 7, 9) but not with the Mut probe (lanes 4, 6, 8, 10). These data show that AcNAC1–4 TFs specifically bound to the wildtype version of this region (27 bp) in accordance with the promoter deletion results and required the TATACGTATA palindromic sequence for binding. Together, the promoter activation and EMSA results support direct NAC activation of the *AcACS1* promoter at a binding site between 389 and 378 bp upstream of the ATG whilst the promoter activation data suggest that additional binding sites for AcNAC4 and AcEIL exist in the proximal region.

## Discussion

### System-1 and -2 regulation

Comparing two different organs in kiwifruit provided a contrast in how ethylene system-1 and system-2 biosynthesis are transcriptionally regulated. During both immature fruit wounding (system-1) and autocatalytic ethylene ripening (system-2) in kiwifruit, ACS activity appears to be predominantly controlled by transcriptional induction of a single gene, *AcACS1*, which preceded peak wound ethylene levels (12 h) and is also highly induced in ripe fruit, during autocatalytic ethylene production (Fig. 4). In kiwifruit leaf wounding, ethylene was also produced but much more rapidly, peaking at 3 h, and surprisingly without an associated increase in expression of any of the ACS genes (Fig. 6 and Supplemental Table S1). *AcACS1* and *AcACS2* are the only two genes expressed in unwounded/wounded leaves, suggesting that these two genes are involved in leaf ethylene production, but neither show an increase in transcription upon wounding (Fig. 6, Supplemental Table S1). The rapid increase in ethylene biosynthesis in wounded kiwifruit leaves appeared to be mostly regulated at a post-transcriptional level. The expression of ACO or NAC genes also changed little during the leaf wounding time course (Fig. 6 and Supplemental Table S1). In tomato, post-transcriptional regulation of SlACS2 after wounding has been shown [63]. During late fruit ripening (system-2 ethylene biosynthesis), transcriptional up-regulation of *AcACS1* in kiwifruit, and *SlACS2* and *4* in tomato are important regulatory steps, but phosphorylation of the proteins is likely an important additional mechanism to achieve the high levels of ACS enzyme activity required during the autocatalytic phase and may be involved in wound responses too.

At least three different types of ACS proteins have been identified based on presence or absence of the C-terminal CDPK phosphorylation site and ETO1 interaction signatures [57] (Fig. 3; S1). While *SlACS1A*, *2* and *6* as well as *AcACS2* all belong to type I (phosphorylated by CDPK), the dominantly expressed *AcACS1* gene in kiwifruit is type II associated with post-translational control by an ETO1-dependent activity inhibition and 26S proteasome degradation. While *SlACS4* also clusters with type I, it is missing key C-terminal characteristic residues (Supplemental Fig. S1 - RLSF/SLSF motif), so may act like a type III ACS. Research on *AtACS7* regulation (type III) has uncovered a ring-type E3 ligase, XBAT32, that plays a role in the regulation of type III and type II ACS protein stability via targeting to the 26S proteasome pathway [64].

The regulation of system-1 and -2 ethylene production is likely to be associated with other hormones. Analysis of the *AcNAC1–4* and *AcACS1, 2* promoters from *A. chinensis* identified several upstream putative MYC binding sites (Supplemental Fig. S3) that may play a role in JA signalling [65]. In *A. deliciosa*, methyl-JA treatment enhanced ethylene induced ripening, which correlated with increased induction of *AdNAC2*, *3* and downstream *AdACS1*, − *2* [66] and rapid wound induction of JA within minutes has been widely reported in plants. A putative NAC binding site was identified in silico/in vitro approximately 2.2 kb upstream in the *AdACS1* promoter but not confirmed by promoter deletion analysis *in planta* [66]. There are further reports that the JA intermediate cis-OPDA may also have distinct signaling activity of its own [67, 68]. In tomato, *SlAREB1* transcriptional activation of *SlNOR* is involved in abscisic acid-modulated ethylene biosynthesis during tomato fruit ripening and may provide another early hormonal link between wounding and NAC expression [69].

### Transcriptional control of ACS

In kiwifruit, several NAC TFs are strongly up-regulated during fruit ripening and induce terpene synthesis [52]. We showed that *AcNAC2–4* are induced at lower levels during fruit wounding and that the amplitude of *AcNAC2–4* induction correlated with *AcACS1* expression levels both during fruit wounding (lower peak expression) and ripening (highest expression), while no induction of NAC TFs and *AcACS1* was observed during leaf wounding (Fig. 6 and Supplemental Table S2). This is not the only described mechanism for regulating kiwifruit ripening. In *A. deliciosa*, *AdNAC6* and *7* have been shown to regulate the *AdACS1* and *AdACO1* promoter. When a miRNA binding site present in the 3′-end of these NAC genes was ablated, they were able to induce promoter activity, suggesting that miRNA levels may influence ripening through affecting NAC mRNA function [70].

In tomato, during fruit ripening and system-2 ethylene production, ACS and ACO transcription are under complex control of TFs such as *SlHB1*, which can bind to the homeobox cis-elements of the tomato *SlACO1* promoter and regulate gene expression in developing fruit [71]. The transcription factor Ripening Inhibitor (*RIN*) also modulates the expression of *SlACS2* by binding to the CArG motif during fruit ripening [72] and was shown to interact with the promoter region of *SlACS4* [73]. Martel et al. (2011) [74] showed a significant correlation between *SlRIN* expression, SlRIN abundance, and *SlACS2* expression. However, recent work has shown that SlRIN is likely to act redundantly with other SEP-like genes to activate the ACS genes and its role has been re-evaluated [49, 75]. *SlERF2/TERF2* are representatives of another class of TFs (ERF/AP2 domain) that specifically interact with the GCC-box of the *NtACS3* tobacco promoter in vitro and in vivo [76].

In tomato, several NAC TFs have also been implicated in regulation of ethylene biosynthesis. The NAC TF tomato mutant *Nor* is caused by a mutation in the *SlNOR* gene [74, 77, 78], but the influence by wildtype NOR is likely less pronounced [50]. Knockouts of a closely related gene *SlNAC3* (*NOR-like1*) also delayed fruit ripening and affected seed development [79, 80]. Overexpression of a third NAC TF *SlNAC1* inhibited fruit ripening by interacting with the regulatory region present in the promoter of ethylene biosynthesis genes (*SlACS2* and *SlACO1*) as shown by yeast one-hybrid [81]. The tomato NAC gene *SlNAC4* has also been implicated in regulating ripening. In *SlNAC4* RNAi fruit, expression of *SlACS2* and *4* as well as *SlACO1* and *3* were repressed during ripening, although no evidence was presented for direct promoter interaction [82].

Respiratory climacteric related ethylene production appears to be controlled by different mechanisms in kiwifruit and tomato (Fig. 1). In kiwifruit, only one ACS gene (*AcACS1*) appeared to be most associated with both fruit system-1 and system-2 ethylene production. This suggests that *AcACS1* was tightly regulated with different complexes controlling the system-1 and system-2 responses in fruit, but other ACS genes are likely involved in other types of ethylene biosynthesis, such as during various stresses and flower petal scenescence. In Arabidopsis for example, all of the twelve ACS genes display different expression patterns throughout growth and development, and during various stress conditions, while in tomato only four out of the nine ACS genes are expressed in fruit [83, 84]. The slower progression of ripening resulting in climacteric ethylene in kiwifruit (Fig. 1) may point to the system-1 response needing to be overcome before autocatalytic ethylene can be produced. In tomato, two sets of ACS genes (*SlACS4* and *2*) are associated with the climacteric phase. *SlACS2* is regulated at the chromatin level through accessibility, DNA methylation and histone modification [46, 85], while *SlACS1A* and *6* are linked with system-1.

## Conclusions

After mechanical wounding a complex array of rapid local and distal signalling events, as well as hormonal and cellular responses are induced. By comparing the wounding response in kiwifruit in fruit and leaves, this work has uncovered key NAC transcriptional regulatory mechanisms involved in system-1 (wound-induced) and system-2 (autocatalytic ripening) ethylene biosynthesis in fruit. In kiwifruit, there appears to be a direct link between NAC TF (*AcNAC2–4*) and *AcACS1* transcript levels in fruit, while in leaves, post-transcriptional/−translational mechanisms are more likely involved in inducing wound ethylene in a more rapid fashion.

## Methods

### Plant material and wound ethylene measurements

Immature *A. chinensis* var. *chinensis* ‘Hort16A’ fruit were harvested from the orchard at The New Zealand Institute for Plant and Food Research Limited (PFR), Riwaka, New Zealand at ~ 110 DAFB/BBCH 78 or ~ 80% of final fruit weight and then kept at room temperature. *A. chinensis* ‘Hort16A’ leaf material (young expanding leaves ~ 10 cm in length) were obtained in November from potted plants grown under ambient temperature and light in a PFR greenhouse at the Mt Albert Research Centre, Auckland, New Zealand. Eating ripe *A. chinensis* ‘Hort16A’ fruit were also obtained from PFR, Riwaka, New Zealand. For the BBCH scale [29], flowering commences at BBCH 60, fruit development at BBCH 70, while fruit maturation and ripening occurs from BBCH 80.

*A. chinensis* ‘Hort16A’ fruit were wounded using a box cutter by making two or four opposing 5 mm deep longitudinal incisions from the distal to proximal (pedicel) end of the fruit. After wounding, fruit were sampled over a 48 h time course (0, 1, 2, 6, 12, 24, 48 h after wounding) for ethylene and RNA extraction. Analysis of variance (ANOVA) was performed separately on the four cuts and two cuts group to identify significant ethylene production differences between time points and time zero. Ethylene measurements were log transformed before modelling to adjust for unequal variance between different time points. R version 3.5.1 [86], R packages nlme (version 3.1–137) and emmeans (version 1.3.4) were used to construct contrasts [87, 88].

Wedges 1 cm wide surrounding the cut and including skin were harvested at the times shown and snap frozen in liquid nitrogen for RNA extractions. For leaf wounding, detached leaves (excluding petiole) were perforated with a 96-well pin tool (containing 2.5 mm diameter flat steel pins of 3 cm in length) immediately after detachment and sampled over a 6 h time course. Ethylene measurements were performed using an ETD-300 ethylene detector with valve control box (Sensor Sense, The Netherlands). Measurements were carried out in 0.75 L or 1.5 L sealed jars at a continuous flow rate of 2 L h^− 1^ filtered (dried and CO_2_ scrubbed) air. Fruit firmness (kgF) was measured using an Effegi hand-held penetrometer (Facchini, Alfonsine, Italy) with 7.9 mm probe while SSC (% brix) was measured using an electronic Atago PAL-1 refractometer (Tokyo, Japan). Soluble solids concentration (SSC) and firmness changes were measured at 120 h after wounding.

### Protein identification, alignments and phylogenetic analysis

Kiwifruit ACS, ACO and NAC TFs were identified by BLASTP searching (cutoff e^− 1^) of the *A. chinensis* ‘Red5’ genome [56]. Amino acid alignments were performed with the Geneious Muscle alignment tool using default parameters and 25 maximum iterations (www.geneious.com), then manually curated. Phylogenetic relationships were assessed using Geneious Tree builder (www.geneious.com) with the Jukes-Cantor Genetic Distance Model [89], UPGMA tree build method [90] and 1000 bootstrap re-samplings using a minimum 50% support threshold.

### Quantitative reverse transcription PCR (qRT-PCR) analysis

Total RNA was extracted by a combination of the “Pine Tree” method [91] and the Spectrum Plant Total RNA kit (Sigma-Aldrich, USA). In short, 100 mg of frozen and ground tissue was mixed with 650 μl “Pine Tree” extraction buffer and incubated at 65 °C for 5 min while shaking. The liquid was then extracted once with an equal volume of chloroform:isoamyl alcohol 24:1. The aqueous phase was transferred to the filtration column of the Spectrum RNA kit followed by RNA purification according to the manufacturer’s instructions. After DNaseI treatment of the total RNA, first-strand cDNA synthesis and expression analysis with gene-specific primers (Supplemental Table S3) were performed on a LightCycler 480 platform (Roche, USA) using SYBR Green Master Mix as described previously [52] using the Transcriptor First Strand cDNA Synthesis Kit (Roche, USA). Samples were normalized against PP2A after evaluation of four reference genes (EF1α, UBC9, PP2A and Actin) [92–95] using GeNorm [96] and BestKeeper [97] based on the combined fruit and leaf data set. (Supplemental Table S4). Expression calculations incorporated the primer efficiencies (E) that were determined based on serial dilutions of the template (See Supplemental Table S3 for reference primers). The data were analyzed using the Target/Reference ratio calculated with the LightCycler® 480 software 1.5, (Supplemental Table S1) using the following equation: Ratio = E (_Ref_)^Cq sample^ / E (_target_)^Cq sample^, Ratio calibrated (CAL) = Ratio ÷ (E (_Ref_)^Cq calibrator^ / E (_target_)^Cq calibrator^). The calibrator for a given target is defined in this study as the biological replicate in the combined fruit and leaf dataset with the highest target gene expression (lowest target Cq). For statistical analysis the gene expression data were log transformed to adjust for unequal variance among treatment groups, then data were fitted to mixed models. Treatment was fitted as a fixed effect, replicate was fitted as a random effect. Contrasts between non- and wounded samples at each time point were made to identify significant differences. All analyses were carried out using R version 3.5.1 [86]. R packages nlme (version 3.1–137) and emmeans (version 1.3.4) were used to perform mixed models and contrasts respectively [87, 88].

### Transient expression promoter analysis by luciferase assays

The 1 kb promoter and smaller fragments of *AcACS1* including the 5′-untranslated region (5′-UTR) were amplified from *A. chinensis ‘*Hort16A’ into the *NcoI*/ATG start site of pGII0800-LUC [62] using primers listed in Supplemental Table S3. NAC and EIL complete TF open reading frames (ORFs) were cloned from ripe fruit cDNA obtained from *A. chinensis ‘*Hort16A’ using primers listed in Supplemental Table S3 into the CaMV 35S-promoter driven pHEX2 vector [62]. Promoter activation was assessed by comparison of firefly (*Photinus pyralis*) luciferase: renilla (*Renilla reniformis*) luciferase luminescence ratios (LUC/REN) determined 3–4 d after *N. benthamiana* infiltration [62]. Promoter to TF ratios of 1:4 were used as described [52].

### Electrophoretic mobility shift assay

The DNA binding domain (182 N-terminal amino acids from each NAC TF) was blunt cloned in frame behind the Maltose Binding Protein purification tag (MBP) in the vector pMAL-c2x (New England Biolabs, USA) using the *XmnI* site and *BamHI* restriction sites (for primers see Supplemental Table S3). Proteins were expressed in DH5α *E. coli* cells and purified according to manufacturer’s instructions (New England Biolabs, USA) using amylose resin and eluted in column buffer [20 mM Tris-HCl pH 7.4, 0.2 M NaCl, 1 mM EDTA and 10 mM maltose]. For the binding assay, ~ 2.5 μg of recombinant NAC protein was mixed with 0.9 pmol of double-stranded 3′-biotinylated DNA probe (EMSA probes; Supplemental Table S3) in binding buffer [0.2 mM dithiothreitol, 0.02 mM EDTA, 5 mM HEPES-KOH, pH 7.6, 30 mm sodium chloride, 0.8 μg of salmon sperm DNA (sheared), and 0.2 μg of poly (dI-dC)] in a 20 μL reaction at room temperature for 15 min. The bound complexes were resolved by electrophoresis on native 4% (w/v) polyacrylamide gels in 0.5% (w/v) tris-borate EDTA buffer containing 5% (v/v) glycerol, pH 8.3, at 200 V for 25 min at 4 °C. The gels were electroblotted onto positively charged Hybond N+ membrane (GE Healthcare; 25 V/15 min) and cross-linked using a UVC500 (Hoefer) at 120 mJ cm^− 2^. Blots were blocked in 1x casein blocking solution (Sigma Aldrich, USA, #B6429) for > 30 min and incubated with 1:2000 Streptactin-HRP (Bio-Rad, USA) for 1 h in blocking buffer and washed according manufacturer’s instructions (Sigma Aldrich, USA). Imaging was conducted with ECL Select substrate (GE Healthcare) using a ChemiDoc MP imager (Bio-Rad).

## Supplementary Information

**Additional file 1: Table S1** qRT-PCR results for ACO, ACS and NAC TF genes in *A. chinensis* ‘Hort16A’ fruit and leaves. **Table S2** Putative NAC transcription factors identified in the *A. chinensis* ‘Red5’ genome. **Table S3** Primer sequences for qRT-PCR, EMSA and cloning. **Table S4** Reference gene validation metrics using GeNorm and BestKeeper. **Fig. S1** Amino acid alignment of the C-terminus of ACS proteins from Arabidopsis, tomato and kiwifruit. **Fig. S2** Electrophoretic mobility shift assays (EMSA) of *AcACS1* promoter fragments (27 bp) with recombinant NAC1–4 proteins. Bottom blot: Original image of Fig. 8. Top blot: EMSA with double the probe concentrations. **Fig. S3** Potential transcription factor binding sites in the *AcACS1*, *AcACS2* and *AcNAC1–4* promoters

## Data Availability

All data generated or analyzed during this study are included in this published article and its supplementary information files.

## Abbreviations

ABA: Abscisic acid
ACC: 1-Aminocyclopropane-1-carboxylic acid
ACS: ACC synthase
ACO: ACC oxidase
AT: Aminotransferase
BBCH: Biologische Bundesanstalt, Bundessortenamt und Chemische Industrie
DAFB: Days after full bloom
EIN3: Ethylene-insensitive3
EMSA: Electrophoretic mobility shift assay
JA: Jasmonic acid
MTA: 5′-Methylthioadenosine
NAC: NAM, ATAF1/2, CUC2
Nor: Non-ripening
qRT-PCR: Quantitative Reverse Transcription Polymerase Chain Reaction
RIN: Ripening inhibitor
ROS: Reactive oxygen species
SAM: S-adenosylmethionine
TF: Transcription factor
