# Supplementary material for: Regulation of wound ethylene biosynthesis by NAC transcription factors in kiwifruit
Source: BMC Plant Biol. 2021 Sep 8;21:411. doi: 10.1186/s12870-021-03154-8 (PMC8425125; doi:10.1186/s12870-021-03154-8)
Supplement: Supplementary file 1 — Additional file 1: Table S1 qRT-PCR results for ACO, ACS and NAC TF genes in A. chinensis ‘Hort16A’ fruit and leaves. Table S2 Putative NAC transcription factors identified in the A. chinensis ‘Red5’ genome. Table S3 Primer sequences for qRT-PCR, EMSA and cloning. Table S4 Reference gene validation metrics using GeNorm and BestKeeper. Fig. S1 Amino acid alignment of the C-terminus of ACS proteins from Arabidopsis, tomato and kiwifruit. Fig. S2 Electrophoretic mobility shift assays (EMSA) of AcACS1 promoter fragments (27 bp) with recombinant NAC1–4 proteins. Bottom blot: Original image of Fig. 8. Top blot: EMSA with double the probe concentrations. Fig. S3 Potential transcription factor binding sites in the AcACS1, AcACS2 and AcNAC1–4 promoters [file 12870_2021_3154_MOESM1_ESM.docx]

**Supplementary material**

Table S1: qRT-PCR results for ACO, ACS and NAC TF genes in *A.* *chinensis* ‘Hort16A’ fruit and leaves

Table S2: Putative NAC transcription factors identified in the *A. chinensis* ‘Red5’ genome

Table S3: Primer sequences for qRT-PCR, EMSA and cloning

Table S4. Reference gene validation metrics using GeNorm and Bestkeeper.

Figure S1: Amino acid alignment of the C-terminus of ACS proteins from Arabidopsis, tomato and kiwifruit

Figure S2: Electrophoretic mobility shift assays (EMSA) of *AcACS1* promoter fragments (27 bp) with recombinant NAC1-4 proteins. Bottom blot: Original image of Figure 8. Top blot: EMSA with double the probe concentrations

Figure S3: Potential transcription factor binding sites in the *AcACS1*, *AcACS2* and *AcNAC1-4* promoters

**Table S1**: qRT-PCR results for ACO, ACS and NAC TF genes in *A.* *chinensis* ‘Hort16A’ fruit and leaves. Data are means from three biological repeats harvested per time point snap frozen for RNA extraction. Data are expressed as a ratio compared to the PP2A reference gene [1]. Primer amplification efficiencies (E) are given in Table S3. ND: Not detected (Cq > 40). Ratio = E(_Ref_)^Cq sample^ / E(_target_)^Cq sample^, Ratio calibrated (CAL) = Ratio ÷ (E(_Ref_)^Cq calibrator^ / E(_target_)^Cq calibrator^ ). Source: Roche Lightcycler Relative Quantification, 2001. Across the combined fruit and leaf data set the calibrator sample is defined in this study as the replicate with the highest expression (lowest target Cq).

| **FRUIT** | | **Treatment: hours after fruit wounding (h) with 2 cuts versus ripe fruit** | | | | | | | |
| --- | --- | --- | --- | --- | --- | --- | --- | --- | --- |
| Gene | Measure | Uncut | S2-1h | S2-2h | S2-6h | S2-12h | S2-24h | S2-48h | Ripe |
| AcACO1 | RATIO | 4.475 | 4.295 | 4.360 | 8.940 | 18.372 | 8.011 | 2.070 | 257.157 |
|  | CAL | 0.016 | 0.016 | 0.016 | 0.032 | 0.067 | 0.029 | 0.008 | 0.935 |
|  | SD | 0.006 | 0.003 | 0.006 | 0.003 | 0.023 | 0.014 | 0.005 | 0.084 |
| AcACO3 | RATIO | 0.803 | 1.183 | 1.281 | 6.542 | 15.610 | 10.737 | 3.555 | 243.311 |
|  | CAL | 0.003 | 0.004 | 0.004 | 0.021 | 0.051 | 0.035 | 0.012 | 0.797 |
|  | SD | 0.000 | 0.001 | 0.001 | 0.003 | 0.018 | 0.016 | 0.005 | 0.187 |
| AcACO4 | RATIO | 0.291 | 0.252 | 0.511 | 1.739 | 1.060 | 0.329 | 0.195 | 0.362 |
|  | CAL | 0.043 | 0.038 | 0.076 | 0.260 | 0.158 | 0.049 | 0.029 | 0.054 |
|  | SD | 0.013 | 0.012 | 0.014 | 0.043 | 0.054 | 0.014 | 0.011 | 0.010 |
| AcACO5 | RATIO | 0.239 | 0.088 | 0.145 | 0.684 | 2.645 | 1.963 | 1.388 | 3.694 |
|  | CAL | 0.002 | 0.001 | 0.001 | 0.007 | 0.027 | 0.020 | 0.014 | 0.037 |
|  | SD | 0.001 | 0.000 | 0.000 | 0.000 | 0.004 | 0.003 | 0.007 | 0.011 |
| AcACO6 | RATIO | 0.605 | 0.161 | 0.152 | 0.119 | 0.501 | 0.236 | 0.276 | 0.120 |
|  | CAL | 0.021 | 0.006 | 0.005 | 0.004 | 0.017 | 0.008 | 0.010 | 0.004 |
|  | SD | 0.011 | 0.001 | 0.001 | 0.001 | 0.002 | 0.003 | 0.005 | 0.002 |
| AcACO7 | RATIO | 0.007 | 0.006 | 0.010 | 0.009 | 0.112 | 0.031 | 0.015 | 1.244 |
|  | CAL | 0.003 | 0.003 | 0.005 | 0.004 | 0.051 | 0.014 | 0.007 | 0.569 |
|  | SD | 0.002 | 0.002 | 0.002 | 0.001 | 0.008 | 0.008 | 0.003 | 0.373 |
| AcACO8 |  | *ND* | *ND* | *ND* | *ND* | *ND* | *ND* | *ND* | *ND* |
| AcACO9 |  | *ND* | *ND* | *ND* | *ND* | *ND* | *ND* | *ND* | *ND* |
| AcACO10 |  | *ND* | *ND* | *ND* | *ND* | *ND* | *ND* | *ND* | *ND* |
| AcACS1 | RATIO | 0.001 | 0.386 | 0.487 | 1.329 | 0.346 | 0.014 | 0.009 | 9.703 |
|  | CAL | 0.000 | 0.027 | 0.034 | 0.093 | 0.024 | 0.001 | 0.001 | 0.678 |
|  | SD | 0.000 | 0.009 | 0.013 | 0.023 | 0.010 | 0.000 | 0.001 | 0.280 |
| AcACS2 | RATIO | 0.001 | 0.047 | 0.033 | 0.017 | 0.008 | 0.004 | 0.004 | 0.030 |
|  | CAL | 0.005 | 0.365 | 0.251 | 0.130 | 0.065 | 0.034 | 0.032 | 0.233 |
|  | SD | 0.002 | 0.143 | 0.120 | 0.007 | 0.008 | 0.003 | 0.014 | 0.115 |
| AcACS6 |  | *ND* | *ND* | *ND* | *ND* | *ND* | *ND* | *ND* | *ND* |
| AcACS8 |  | *ND* | *ND* | *ND* | *ND* | *ND* | *ND* | *ND* | *ND* |
| AcACS9 |  | *ND* | *ND* | *ND* | *ND* | *ND* | *ND* | *ND* | *ND* |
| AcACS10 |  | *ND* | *ND* | *ND* | *ND* | *ND* | *ND* | *ND* | *ND* |
| AcACS11 |  | *ND* | *ND* | *ND* | *ND* | *ND* | *ND* | *ND* | *ND* |
| AcACS12/R |  | *ND* | *ND* | *ND* | *ND* | *ND* | *ND* | *ND* | *ND* |
| AcACS13/R |  | *ND* | *ND* | *ND* | *ND* | *ND* | *ND* | *ND* | *ND* |
| AcNAC1 | RATIO | 2.846 | 1.699 | 1.390 | 2.634 | 1.753 | 2.043 | 2.417 | 2.629 |
|  | CAL | 0.726 | 0.433 | 0.355 | 0.672 | 0.447 | 0.521 | 0.616 | 0.670 |
|  | SD | 0.207 | 0.186 | 0.186 | 0.068 | 0.048 | 0.246 | 0.361 | 0.172 |
| AcNAC2 | RATIO | 0.920 | 0.327 | 0.373 | 2.945 | 1.181 | 0.681 | 0.676 | 3.427 |
|  | CAL | 0.210 | 0.075 | 0.085 | 0.672 | 0.269 | 0.155 | 0.154 | 0.782 |
|  | SD | 0.109 | 0.033 | 0.073 | 0.118 | 0.037 | 0.061 | 0.072 | 0.193 |
| AcNAC3 | RATIO | 0.283 | 0.137 | 0.141 | 2.722 | 2.228 | 0.461 | 0.367 | 5.867 |
|  | CAL | 0.038 | 0.018 | 0.019 | 0.367 | 0.301 | 0.062 | 0.050 | 0.792 |
|  | SD | 0.014 | 0.007 | 0.011 | 0.040 | 0.113 | 0.035 | 0.032 | 0.193 |
| AcNAC4 | RATIO | 1.416 | 1.736 | 1.187 | 4.656 | 1.373 | 0.508 | 0.447 | 6.283 |
|  | CAL | 0.166 | 0.203 | 0.139 | 0.545 | 0.161 | 0.059 | 0.052 | 0.735 |
|  | SD | 0.123 | 0.156 | 0.062 | 0.048 | 0.037 | 0.015 | 0.019 | 0.232 |
| AcNAC5 | RATIO | 0.077 | 0.031 | 0.053 | 0.259 | 0.159 | 0.045 | 0.032 | 1.938 |
|  | CAL | 0.022 | 0.009 | 0.015 | 0.073 | 0.045 | 0.013 | 0.009 | 0.545 |
|  | SD | 0.011 | 0.005 | 0.006 | 0.014 | 0.009 | 0.005 | 0.000 | 0.395 |
| AcNAC6 | RATIO | 0.234 | 0.050 | 0.044 | 0.521 | 0.299 | 0.077 | 0.056 | 0.575 |
|  | CAL | 0.328 | 0.071 | 0.061 | 0.730 | 0.419 | 0.107 | 0.079 | 0.806 |
|  | SD | 0.132 | 0.056 | 0.005 | 0.062 | 0.108 | 0.066 | 0.010 | 0.185 |

| **LEAVES** | | **Treatment (hours after +/- leaf wounding)** | | | | |
| --- | --- | --- | --- | --- | --- | --- |
| Gene | Measure | T0 | T3h- | T3h+ | T6h- | T6h+ |
| AcACO1 | RATIO | 0.149 | 0.214 | 0.140 | 0.632 | 0.214 |
|  | CAL | 0.001 | 0.001 | 0.001 | 0.002 | 0.001 |
|  | SD | 0.000 | 0.000 | 0.000 | 0.002 | 0.000 |
| AcACO3 | RATIO | 0.408 | 0.507 | 0.532 | 2.145 | 1.052 |
|  | CAL | 0.001 | 0.002 | 0.002 | 0.007 | 0.003 |
|  | SD | 0.001 | 0.000 | 0.001 | 0.005 | 0.002 |
| AcACO4 | RATIO | 0.537 | 0.925 | 1.057 | 2.141 | 3.620 |
|  | CAL | 0.080 | 0.138 | 0.158 | 0.320 | 0.540 |
|  | SD | 0.043 | 0.042 | 0.073 | 0.110 | 0.407 |
| AcACO5 | RATIO | 12.885 | 22.214 | 17.047 | 61.097 | 37.173 |
|  | CAL | 0.131 | 0.225 | 0.173 | 0.620 | 0.377 |
|  | SD | 0.087 | 0.016 | 0.080 | 0.340 | 0.156 |
| AcACO6 | RATIO | 1.283 | 2.747 | 2.323 | 12.199 | 18.312 |
|  | CAL | 0.045 | 0.095 | 0.081 | 0.424 | 0.636 |
|  | SD | 0.023 | 0.018 | 0.045 | 0.198 | 0.318 |
| AcACO7 | RATIO | 0.121 | 0.127 | 0.108 | 0.467 | 0.305 |
|  | CAL | 0.055 | 0.058 | 0.050 | 0.214 | 0.140 |
|  | SD | 0.030 | 0.018 | 0.035 | 0.135 | 0.082 |
| AcACO8 |  | ND | ND | ND | ND | ND |
| AcACO9 |  | ND | ND | ND | ND | ND |
| AcACO10 |  | ND | ND | ND | ND | ND |
| AcACS1 | RATIO | 0.063 | 0.006 | 0.010 | 0.020 | 0.026 |
|  | CAL | 0.004 | 0.000 | 0.001 | 0.001 | 0.002 |
|  | SD | 0.001 | 0.001 | 0.000 | 0.001 | 0.001 |
| AcACS2 | RATIO | 0.106 | 0.035 | 0.062 | 0.069 | 0.034 |
|  | CAL | 0.818 | 0.268 | 0.479 | 0.533 | 0.265 |
|  | SD | 0.161 | 0.118 | 0.059 | 0.278 | 0.109 |
| AcACS6 | RATIO | ND | ND | ND | ND | ND |
| AcACS8 | RATIO | ND | ND | ND | ND | ND |
| AcACS9 | RATIO | ND | ND | ND | ND | ND |
| AcACS10 | RATIO | ND | ND | ND | ND | ND |
| AcACS11 | RATIO | ND | ND | ND | ND | ND |
| AcACS12/R | RATIO | ND | ND | ND | ND | ND |
| AcACS13/R | RATIO | ND | ND | ND | ND | ND |
| AcNAC1 | RATIO | 0.010 | 0.038 | 0.068 | 0.118 | 0.051 |
|  | CAL | 0.003 | 0.010 | 0.017 | 0.030 | 0.013 |
|  | SD | 0.001 | 0.005 | 0.006 | 0.024 | 0.005 |
| AcNAC2 | RATIO | 0.000 | 0.000 | 0.000 | 0.000 | 0.000 |
|  | CAL | 0.000 | 0.000 | 0.000 | 0.000 | 0.000 |
|  | SD | 0.000 | 0.000 | 0.000 | 0.000 | 0.000 |
| AcNAC3 | RATIO | 0.000 | 0.000 | 0.000 | 0.000 | 0.000 |
|  | CAL | 0.000 | 0.000 | 0.000 | 0.000 | 0.000 |
|  | SD | 0.000 | 0.000 | 0.000 | 0.000 | 0.000 |
| AcNAC4 | RATIO | 0.088 | 0.100 | 0.145 | 0.248 | 0.120 |
|  | CAL | 0.010 | 0.012 | 0.017 | 0.029 | 0.014 |
|  | SD | 0.006 | 0.004 | 0.007 | 0.023 | 0.005 |
| AcNAC5 | RATIO | 0.001 | 0.007 | 0.006 | 0.028 | 0.110 |
|  | CAL | 0.000 | 0.002 | 0.002 | 0.008 | 0.031 |
|  | SD | 0.000 | 0.001 | 0.001 | 0.005 | 0.016 |
| AcNAC6 | RATIO | 0.020 | 0.047 | 0.071 | 0.105 | 0.173 |
|  | CAL | 0.027 | 0.066 | 0.099 | 0.147 | 0.242 |
|  | SD | 0.015 | 0.022 | 0.020 | 0.094 | 0.082 |

**Table S2**: The 147 putative NAC transcription factors identified in the *A. chinensis* ‘Red5’ genome [2]. The numbering corresponds to the phylogram shown in Supplemental Figure S3 and previous publications (NAC1-4) [3], Achn169421 [4]. NACs characterised in this work are shown in bold/red.

| **Gene** | **Accession [2]** | **Comment** | **Gene** | **Accession** | **Comment** | **Gene** | **Accession** | **Comment** |
| --- | --- | --- | --- | --- | --- | --- | --- | --- |
| NAC7 | Acc30501.1 |  | NAC51 | Acc02456.1 |  | NAC101 | Acc07174.1 |  |
| NAC8 | Acc05549.1 |  | NAC52 | Acc30600.1 |  | NAC102 | Acc16347.1 |  |
| NAC9 | Acc16199.1 |  | NAC53 | Acc05638.1 |  | NAC103 | Acc07217.1 |  |
| NAC10 | Acc16198.1 |  | NAC54 | Acc31273.1 |  | NAC104 | Acc06334.1 |  |
| NAC11 | Acc01968.1 |  | NAC55 | Acc28543.1 |  | NAC105 | Acc02348.1 |  |
| NAC12 | Acc06991.1 |  | NAC56 | Acc32901.1 |  | NAC106 | Acc30714.1 |  |
| NAC13 | Acc24543.1 |  | NAC57 | Acc14024.1 |  | NAC107 | Acc05742.1 |  |
| NAC14 | Acc24618.1 |  | NAC58 | Acc23299.1 |  | NAC108 | Acc33475.1 |  |
| NAC15 | Acc24581.1 |  | NAC59 | Acc13041.1 |  | NAC109 | Acc26259.1 |  |
| NAC16 | Acc20847.1 |  | NAC60 | Acc18944.1 |  | NAC110 | Acc13969.1 |  |
| NAC17 | Acc16067.1 |  | NAC61 | Acc13874.1 |  | NAC111 | Acc00008.1 |  |
| NAC18 | Acc29981.1 |  | NAC62 | Acc17306.1 |  | NAC112 | Acc26425.1 |  |
| NAC19 | Acc29980.1 |  | NAC63 | Acc09530.1 |  | NAC113 | Acc26602.1 |  |
| NAC20 | Acc19004.1 |  | NAC64 | Acc16948.1 |  | NAC114 | Acc29402.1 |  |
| NAC21 | Acc16066.1 |  | NAC65 | Acc32093.1 |  | NAC115 | Acc33010.1 |  |
| NAC22 | Acc32998.1 |  | NAC66 | Acc30045.1 |  | NAC116 | Acc00767.1 |  |
| NAC23 | Acc31505.1* | Achn169421 [4] | NAC67 | Acc32092.1 |  | NAC117 | Acc10125.1 |  |
| NAC24 | Acc06350.1 |  | NAC68 | Acc30044.1 |  | NAC118 | Acc23463.1 |  |
| NAC25 | Acc21211.1 |  | NAC69 | Acc10940.1 |  | NAC119 | Acc04460.1 |  |
| NAC26 | Acc06757.1 |  | NAC70 | Acc10715.1 |  | NAC120 | Acc22388.1 |  |
| NAC27 | Acc17304.1 |  | NAC71 | Acc06655.1 |  | NAC121 | Acc15482.1 |  |
| NAC28 | Acc09528.1 |  | NAC72 | Acc23678.1 |  | NAC122 | Acc30738.1 |  |
| NAC29 | Acc30177.1 |  | NAC73 | Acc04655.1 |  | NAC123 | Acc05752.1 |  |
| NAC30 | Acc25122.1 |  | NAC74 | Acc28994.1 |  | NAC124 | Acc29877.1 |  |
| NAC31 | Acc17303.1 |  | NAC75 | Acc28139.1 |  | NAC125 | Acc18287.1 |  |
| NAC32 | Acc09527.1 |  | NAC76 | Acc15892.1 |  | NAC126 | Acc04082.1 |  |
| NAC33 | Acc00753.1 |  | NAC77 | Acc02576.1 |  | NAC127 | Acc02334.1 |  |
| NAC34 | Acc10114.1 |  | NAC78 | Acc20367.1 |  | NAC128 | Acc33448.1 |  |
| **NAC5** | **Acc17357.1** | **SlNOR-like** | NAC79 | Acc03232.1 |  | NAC129 | Acc19292.1 |  |
| **NAC6** | **Acc09579.1** | **SlNOR-like** | NAC80 | Acc32083.1 |  | NAC130 | Acc14650.1 |  |
| **NAC1** | **Acc13706.1** | **SlNOR-like [3]** | NAC81 | Acc21503.1 |  | NAC131 | Acc25630.1 |  |
| **NAC2** | **Acc26399.1** | **SlNOR-like [3]** | NAC82 | Acc30034.1 |  | NAC132 | Acc32037.1 |  |
| **NAC3** | **Acc21700.1** | **SlNOR-like [3]** | NAC83 | Acc33687.1 |  | NAC133 | Acc29991.1 |  |
| NAC35 | Acc30379.1 |  | NAC84 | Acc04951.1 |  | NAC134 | Acc24162.1 |  |
| NAC36 | Acc30380.1 |  | NAC85 | Acc24239.1 |  | NAC135 | Acc26028.1 |  |
| NAC37 | Acc19476.1 |  | NAC86 | Acc05335.1 |  | NAC136 | Acc14881.1 |  |
| NAC38 | Acc13705.1 |  | NAC87 | Acc26558.1 |  | NAC137 | Acc19734.1 |  |
| NAC39 | Acc08011.1 |  | NAC88 | Acc21792.1 |  | NAC138 | Acc08328.1 |  |
| NAC40 | Acc05460.1 |  | NAC89 | Acc08058.1 |  | NAC139 | Acc15513.1 |  |
| NAC41 | Acc12299.1 |  | NAC90 | Acc05484.1 |  | NAC140 | Acc24464.1 |  |
| NAC42 | Acc05057.1 |  | NAC91 | Acc27590.1 |  | NAC141 | Acc05200.1 |  |
| NAC43 | Acc24103.1 |  | NAC92 | Acc24657.1 |  | NAC142 | Acc19912.1 |  |
| NAC44 | Acc32712.1 |  | NAC93 | Acc25396.1 |  | NAC143 | Acc15781.1 |  |
| NAC45 | Acc14317.1 |  | NAC94 | Acc20922.1 |  | NAC144 | Acc28908.1 |  |
| NAC46 | Acc06142.1 |  | NAC95 | Acc25397.1 |  | NAC145 | Acc16760.1 |  |
| **NAC4** | **Acc08288.1** | **[3]** | NAC96 | Acc20923.1 |  | NAC146 | Acc27956.1 |  |
| NAC47 | Acc04398.1 |  | NAC97 | Acc27591.1 |  | NAC147 | Acc03559.1 |  |
| NAC48 | Acc23760.1 |  | NAC98 | Acc24658.1 |  |  |  |  |
| NAC49 | Acc29633.1 |  | NAC99 | Acc01100.1 |  |  |  |  |
| NAC50 | Acc17988.1 |  | NAC100 | Acc10851.1 |  |  |  |  |

**Table S3**: Primer sequences for qRT-PCR, EMSA and cloning.

| **Accession [2]** | **Gene** | **qRT-PCR Primer forward** | **qRT-PCR Primer reverse** | **Efficiency** | **Amplicon (bp)** |
| --- | --- | --- | --- | --- | --- |
| **Kiwifruit** | | | | | |
| Acc24995.1 | AcACO1 | CATTGTTACAGGAGCGCCGCTT | CCAAGCAAACCCTAAACATGCGAA | 2 | 109 |
| Acc20538.1 | AcACO3 | TGTGTGCTTGGCTAGTGTGTGTGAA | CCATTAACACCACTGCAACCAGACA | 1.947 | 107 |
| Acc09717.1 | AcACO4 | CGCTTTTACGTGTCCCTGAGGCTA | GCAAATCCTGTGGTCAGCACATCA | 2 | 110 |
| Acc13619.1 | AcACO5 | GCAACAGCTTAATTAAGATTCGATGC | ACTTGACACTTCCCCTAAACCAA | 1.954 | 70 |
| Acc17490.1 | AcACO6 | CGTGTGCTTTTACATGTCCCAGAGA | TCATGAAAACCACCACAACCAGACA | 1.965 | 142 |
| Acc19252.1 | AcACO7 | GTCGATGCAAAGAGAACAGGAAT | ACGAACCCAACAATTTCCCTTTT | 2 | 88 |
| Acc24093.1 | AcACO8 | TCTCCAAAGAAGTGTAGAAGTGGA | AGGCACCCAATATTACACGGG | ND* | 83 |
| Acc23323.1 | AcACO9 | TCTCTTGTTTAGCCCTCTTTGCA | TGTGTTCCAACAAAGTTCAAACAGT | ND | 93 |
| Acc05046.1 | AcACO10 | GCTCATCACTCTCCGAAGAAGT | AGCACCCAATATTACACAGGAGA | ND | 110 |
| Acc05955.1 | AcACS1 | CCAACAACAACCATAGTCAGAGAAT | GCGCCCGTCGAATGATAATC | 1.819 | 98 |
| Acc15646.1 | AcACS2 | TGAGACGGCACTGTGGAGGCTA | CAGCCAGGGTCAGAGCAATGAAA | 1.968 | 87 |
| Acc12100.1 | AcACS6 | CAGTGGGCTATTTTGTTGGGTTGAT | CCCCAACCTCGAAAAGCAACTTT | ND | 101 |
| Acc19838.1 | AcACS8 | TGGAATCAGCTGTTTGAATGGCAA | ATTACGCGCCACAGCTCCATTT | ND | 108 |
| Acc30932.1 | AcACS9 | CGCCCGGTTTGTCGTGTCAT | GCGAGATCCAAGGTGTCTTCAGTCA | 1.941 | 85 |
| Acc09164.1 | AcACS10 | GCTATGGCGCGGCATAATCAA | CCAGGCTCTGTGCAGTGAAAAGAA | ND | 75 |
| Acc20046.1 | AcACS11 | CGAGATGGCATTGTGGCAGCTA | TGGCTCGGAGCAATGAAAGGAT | ND | 82 |
| Acc33667.1 | AcACS12 | ATTCACTGTGACAGTTCCAACAAT | CGAGGATATGTTCTAGGGTTGACC | ND | 166 |
| Acc33679.1 | AcACS12R | CGATCCACTGTGACAGTTCTAAC | AGGATTTGTTCTAGGGTTGACCG | ND | 166 |
| Acc13019.1/Acc13020.1.1 | AcACS13/13R | GTAGGCGGGCTGAGGAATG | ATCGCTTCCCAAACAGCCAA | ND | 128 |
| Acc13706.1 | AcNAC1 | AAGAGCAACCTCCCAAGACCAA | GAAGGTGGCATTCTTGCAAGCAT | 1.971 | 77 |
| Acc26399.1 | AcNAC2 | CCGGTGGCGATCATAGCTGAAAT | CCGGTTCCTCGGACTGAAAAAAT | 1.963 | 111 |
| Acc21700.1 | AcNAC3 | CAAACCCCAAAACTCCCACCAA | GCTGTGACTGAGCATTTCGTCGAA | 1.96 | 99 |
| Acc08288.1 | AcNAC4 | TAGGACTGATTGGATCATGCACGAA | CGGCAAAGAACCCAATTCTCCATA | 1.96 | 99 |
| Acc17357.1 | AcNAC5 | CATTGGATCAAAGGGATTTCAG | CGGTGGGAACTGATTGAG | 1.851 | 82 |
| Acc09579.1 | AcNAC6 | CGATGGATCAAAGGGGTTTC | CGGCACTTGATTGAGCAA | 1.867 | 79 |
| Acc01971.1 | EF1a | GCACTGTCATTGATGCTCCT | CCAGCTTCAAAACCACCAGT | 1.968 | 120 |
| Acc05529.1/Acc08081.1/  Acc08082.1 | Actin | CCAAGGCCAACAGAGAGAAG | GACGGAGGATAGCATGAGGA | 1.958 | 198 |
| Acc29411.1/Acc33246.1 | PP2A | GCAGCACATAATTCCACAGG | TTTCTGAGCCCATAACAGGAG | 1.886 | 110 |
| Acc01363.1/Acc02645.1 | UBC9 | CCATTTCCAAGGTGTTGCTT | TACTTGTTCCGGTCCGTCTT | 1.995 | 109 |
| *ND: Not determined |  |  |  |  |  |
| **EMSA probes** | **Gene** | **Primer forward** | **Primer reverse** |  |  |
| Acc05955.1 | AcACS1 probe N1-bio-3' | CATTATACGTATAGTCAACCACATAAC | GTTATGTGGTTGACTATACGTATAATG |  |  |
| Acc05955.1 | AcACS1 probe N1MUT-bio-3' | CATCGATCCATCTGTCAACCACATAAC | GTTATGTGGTTGACAGATGGATCGATG |  |  |
|  |  | NAC consensus and mutated site underlined |  |  |  |
| **Maltose BP (pMAL-C2X)** | **Gene** | **Primer forward** | **Primer reverse** |  |  |
| Acc13706.1 | NAC1MBP | ATGGAGAGCACGGATTCG | TTAAGGATCCTCATGGTCGTGGGTGGTT |  |  |
| Acc26399.1 | NAC2MBP | ATGGAGAGCCCGGATTCG | TTAAGGATCCTCAGGCTCTTGGTGGGTT |  |  |
| Acc21700.1 | NAC3MBP | ATGGAGAGCACGGATTCA | TTAAGGATCCTCAGGGTCTTGATGGGTG |  |  |
| Acc08288.1 | NAC4MBP | ATGGAGAAGCTCAACGTTG | TTAAGGATCCTCAATTTTTAGTAC  TTCTTTTCTTCAAAAATA |  |  |
|  |  |  | BamH1 sites underlined |  |  |
| **AcACS1 promoter (pGII0800-LUC)** | **Gene** | **Primer** | **Direction** |  |  |
| Acc05955.1 | AcACS1pro1000F | TTAAGGTACCAAAATACTGTTACGACAAGAGGT | Forward, KpnI |  |  |
| Acc05955.1 | AcACS1pro500F | TTAAGGTACCATTTGTACTTAGTGGATGACACT | Forward, KpnI |  |  |
| Acc05955.1 | AcACS1pro436F | TTAAGGTACCAATACACGTCATATTTGACACTTTC | Forward, KpnI |  |  |
| Acc05955.1 | AcACS1pro389F | TTAAGGTACCATTATACGTATAGTCAACCACA | Forward, KpnI |  |  |
| Acc05955.1 | AcACS1pro374F | TTAAGGTACCAACCACATAACTGGGCA | Forward, KpnI |  |  |
| Acc05955.1 | AcACS1pro350F | TTAAGGTACCTGCAAAATTTAGCAATTTTTATTAAACATGTG | Forward, KpnI |  |  |
| Acc05955.1 | AcACS1pro300F | TTAAGGTACCTTTTATGTTTTACGGCAGATCCT | Forward, KpnI |  |  |
| Acc05955.1 | AcACS1pro250F | TTAAGGTACCAATGCAATTATGTCTATCAGTGTATC | Forward, KpnI |  |  |
| Acc05955.1 | AcACS1pro200F | TTAAGGTACCAGCAGTGAAGATGACGTG | Forward, KpnI |  |  |
| Acc05955.1 | AcACS1proR L3-PHOS-5' | CATGGTTTGCAATTGAGGCCCA | Reverse A, NcoI |  |  |
| Acc05955.1 | AcACS1proR S3 | GTTTGCAATTGAGGCCCA | Reverse B, NcoI |  |  |

**Table S4.** Reference gene validation metrics using GeNorm and BestKeeper.

| **GeNorm:** |
| --- |
| AdEF1α Adactin AdPP2A AdUBC |
| AdEF1α 1.953166 1.351405 1.35203 |
| Adactin 1.953166 1.481904 1.668625 |
| AdPP2A 1.351405 1.481904 0.72075 |
| AdUBC 1.35203 1.668625 0.72075 |
|  |
| M: 1.5522 1.701232 1.184686 1.247135 |
|  |
| **BestKeeper:** |
| AdEF1α Adactin AdPP2A AdUBC |
| n 39 39 39 39 |
| geo Mean [CP] 22.98 25.49 26.93 24.83 |
| ar Mean [CP] 23.03 25.53 26.94 24.84 |
| min [CP] 20.05 23.66 25.12 23.37 |
| max [CP] 26.23 28.95 30.03 27.34 |
| std dev [± CP] 1.24 1.15 0.80 0.75 |
| CV [% CP] 5.39 4.50 2.97 3.02 |
|  |
| **Regression Analysis - HKG vs. BestKeeper :** |
| AdEF1α Cp Adactin Cp AdPP2A Cp AdUBC Cp |
| vs. vs. vs. vs. |
| BK BK BK BK |
| coeff. of corr. [r] 0.749 0.521 0.838 0.732 |
| coeff. of det. [r^2] 0.561 0.271 0.702 0.536 |
| intercept [CP] -9.358 3.514 2.279 4.093 |
| slope [CP] 1.294 0.880 0.985 0.829 |
| SE [CP] ±0.986 ±1.242 ±0.553 ±0.665 |
| p-value 0.001 0.001 0.001 0.001 |
| Power [x-fold] 2.40 1.81 1.88 1.70 |

**
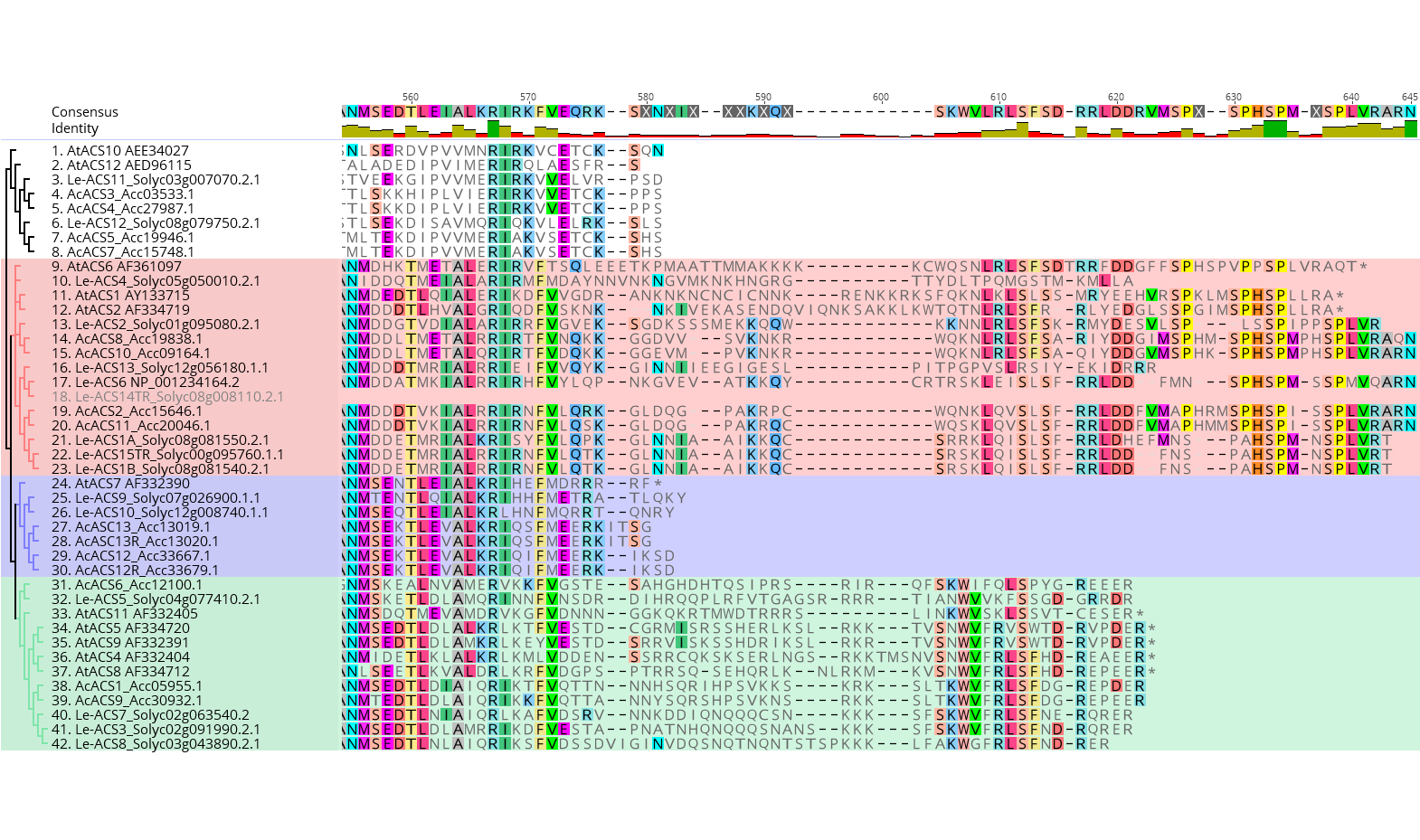
**

**Figure S1.** Amino acid alignment of the C-terminus of aminocyclopropane-1-carboxylic acid synthase (ACS) proteins from Arabidopsis (At), tomato (Le) and kiwifruit (Ac). Boxed: WVF/RLSF motif and RDE rich domain. Type I (red) = RLSF/SLSF only, Type II (green) = WVF, RLSF and RDE rich domains (TOE/target of ETO1 domain), Type III (blue) = absence of type I/II domains (based on Yoshida et al. 2006 [5]). Aminotransferase cluster in white background. TR: likely truncated proteins.


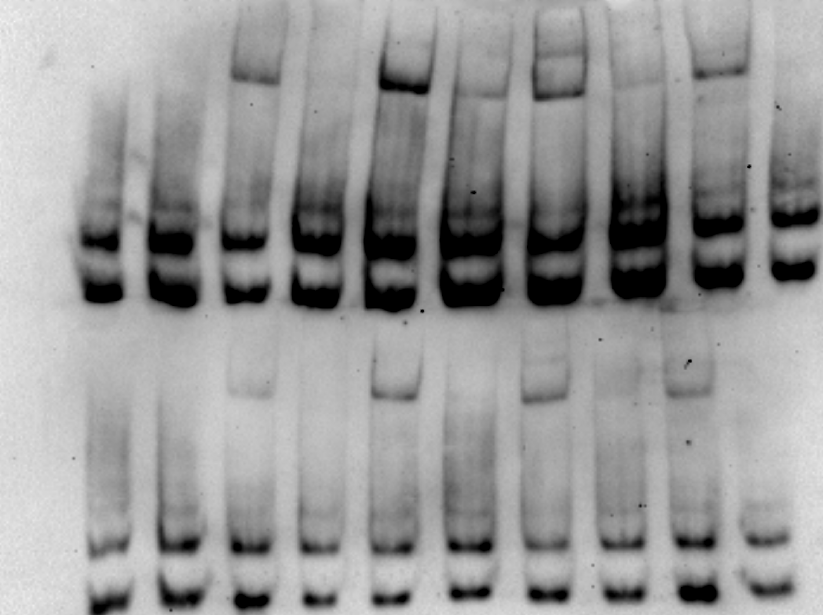


**Figure S2.** Electrophoretic mobility shift assays (EMSA) of *AcACS1* promoter fragments (27 bp) with recombinant NAC1-4 proteins. Bottom blot: Original image of Figure 8. Top blot: EMSA with double the probe concentrations.


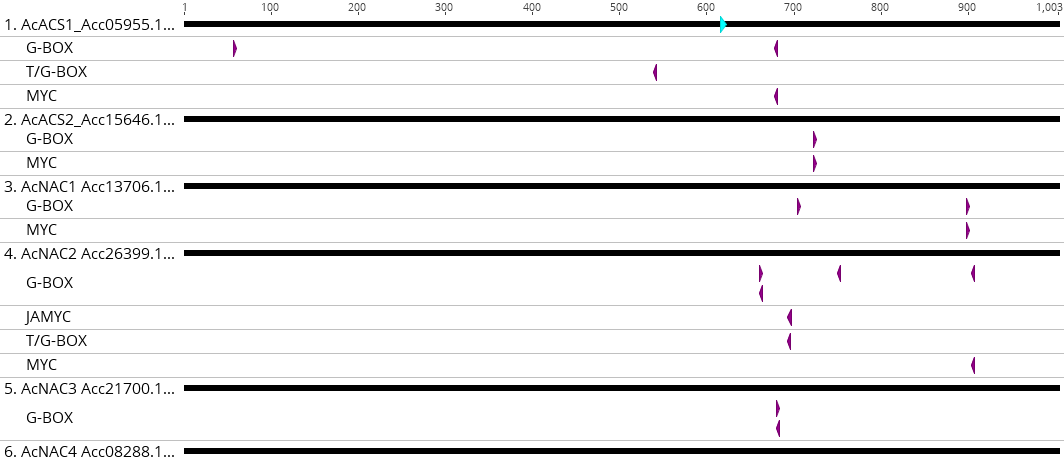


**Figure S3.** Potential transcription factor binding motifs in the *AcACS1*, *AcACS2* and *AcNAC1-4* promoters. Shown is the region 1 kb upstream of the ATG. Light blue: NAC DNA binding site; TATACGTATA. Purple: MYC DNA binding sites; CACATG, G-BOX: CACNTG, T/G-BOX: AACGTG, JAMYC: AAACGTG.

**Supplemental References:**

1. Voogd C, Wang TC, Varkonyi-Gasic E: **Functional and expression analyses of kiwifruit *SOC1*-like genes suggest that they may not have a role in the transition to flowering but may affect the duration of dormancy**. *J Exp Bot* 2015, **66**(15):4699-4710.

2. Pilkington SM, Crowhurst R, Hilario E, Nardozza S, Fraser L, Peng YY, Gunaseelan K, Simpson R, Tahir J, Deroles SC *et al*: **A manually annotated *Actinidia chinensis* var. *chinensis* (kiwifruit) genome highlights the challenges associated with draft genomes and gene prediction in plants**. *BMC Genomics* 2018, **19**(1):257.

3. Nieuwenhuizen NJ, Chen XY, Wang MY, Matich AJ, Perez RL, Allan AC, Green SA, Atkinson RG: **Natural variation in monoterpene synthesis in Kiwifruit: Transcriptional regulation of terpene synthases by NAC and ETHYLENE-INSENSITIVE3-like transcription factors**. *Plant Physiol* 2015, **167**(4):1243-1258.

4. Mitalo OW, Tokiwa S, Kondo Y, Otsuki T, Galis I, Suezawa K, Kataoka I, Doan AT, Nakano R, Ushijima K *et al*: **Low temperature storage stimulates fruit softening and sugar accumulation without ethylene and aroma volatile production in kiwifruit**. *Front Plant Sci* 2019, **10**.

5. Yoshida H, Wang KL, Chang CM, Mori K, Uchida E, Ecker JR: **The ACC synthase TOE sequence is required for interaction with ETO1 family proteins and destabilization of target proteins**. *Plant Molecular Biology* 2006, **62**(3):427-437.
